# Probing the Folding-Unfolding Transition of a Thermophilic Protein, MTH1880

**DOI:** 10.1371/journal.pone.0145853

**Published:** 2016-01-14

**Authors:** Heeyoun Kim, Sangyeol Kim, Youngjin Jung, Jeongmin Han, Ji-Hye Yun, Iksoo Chang, Weontae Lee

**Affiliations:** 1 Department of Biochemistry, College of Life Science and Biotechnology, Yonsei University, Seoul, 120–740, Korea; 2 Department of Physics, Pusan National University, Busan, 609–735, Korea; 3 Center for Proteome Biophysics, Daegu Gyeongbuk Institute of Science and Technology (DGIST), Daegu, 711–873, Korea; 4 Department of Brain and Cognitive Sciences, DGIST, Daegu, 711–873, Korea; Università degli Studi di Milano, ITALY

## Abstract

The folding mechanism of typical proteins has been studied widely, while our understanding of the origin of the high stability of thermophilic proteins is still elusive. Of particular interest is how an atypical thermophilic protein with a novel fold maintains its structure and stability under extreme conditions. Folding-unfolding transitions of MTH1880, a thermophilic protein from *Methanobacterium thermoautotrophicum*, induced by heat, urea, and GdnHCl, were investigated using spectroscopic techniques including circular dichorism, fluorescence, NMR combined with molecular dynamics (MD) simulations. Our results suggest that MTH1880 undergoes a two-state N to D transition and it is extremely stable against temperature and denaturants. The reversibility of refolding was confirmed by spectroscopic methods and size exclusion chromatography. We found that the hyper-stability of the thermophilic MTH1880 protein originates from an extensive network of both electrostatic and hydrophobic interactions coordinated by the central β-sheet. Spectroscopic measurements, in combination with computational simulations, have helped to clarify the thermodynamic and structural basis for hyper-stability of the novel thermophilic protein MTH1880.

## Introduction

Folding mechanisms for a handful of proteins have been examined extensively using various techniques for a long time [1–11]. Previous reports have shown that protein-folding pathways are diverse and highly protein-dependent [12–18]. Knowledge of folding-unfolding transitions between native and denatured conformations served in understanding protein’s biological function [19, 20]. Partially folded structures play an important role during the early stages of refolding that occur prior to the formation of the native state. Urea and guanidine hydrochloride (GdnHCl) are widely used as protein denaturing agents [21–29]. The mechanism how these denaturants disrupt protein structure could be either a direct or an indirect mechanism depending on a protein [30, 31]. Other alternative models for this have been also pursued [32, 33]. Hyper-thermophilic and thermophilic proteins maintain structural stability under extreme conditions, while mesophilic proteins are easily unfolded or denatured [34–36]. Although a number of studies have attempted to uncover the origins of protein stability by probing the structure and thermodynamics of both thermophilic and mesophilic proteins, a molecular mechanistic understanding of hyperstability in thermophilic proteins is still required [35–46].

MTH1880 is a putative thermophilic protein from *Methanobacterium thermoautotrophicum* [47–49]. Solution structures of the thermophilic proteins derived from *M*. *thermoautotrophicum* have been determined by structural genomics project [47, 50, 51, 52]. Among them, we have reported that MTH1880 is a calcium binding protein with a novel motif containing an archetypal α+β motif [52, 53]. Although MTH1880 has been considered as a useful model protein for the folding study of thermophilic protein, little is known about its thermodynamic stability or folding character. Previous studies have examined the folding-unfolding mechanism of mesophilic proteins by circular dichroism, fluorescence spectroscopy, size-exclusion chromatography, and NMR [7, 9, 11, 29, 54]. Molecular dynamics simulations, in particular, played an important role in complementing experimental data for the folding study of mesophilic protein [55, 56, 57]. In this work, we initiated the folding and thermodynamics studies to uncover the origins of hyper-stability and folding of thermophilic protein MTH1880 by both spectroscopic and computational method.

## Materials and Methods

### Cloning, over-expression, and purification of MTH1880

The MTH1880 gene was obtained from *Methanobacterium thermoautotropjicum* genomic DNA by PCR amplification and it was used as a template to clone wild type and seven mutant proteins, K13A (MTH1880^K13A^), V23A (MTH1880^V23A^), D36A (MTH1880 ^D36A^), D38A (MTH1880 ^D38A^), I40A(MTH1880 ^I40A^), I42A(MTH1880 ^I42A^) and V53A (MTH1880 ^V53A^). Each was cloned using BamHI and XhoI sites in the vector pET21b (Novagen), as a fusion protein with an N-terminal hexahistidine affinity tag and TEV cleavage site (ENLYFQG). *Escherichia coli* (Strain BL21 DE3) was the host for non-labeled MTH1880 or ^15^N labeled MTH1880 and were grown in LB and M9 minimal media containing (U-^15^N)at 37°C, and then 1 mM IPTG was added to induce expression for 15 h at 25°C. Cells were harvested by centrifugation and stored at -80°C. Harvested cells were disrupted by sonication in lysis buffer (25 mM sodium phosphate, 300 mM NaCl, and protease inhibitor cocktail (Roche), pH 8.0). The (His)_6_ tag fusion proteins were purified with immobilized metal affinity chromatography on a Ni-NTA column (Amersham Pharmacia) and cleaved by tobacco etch virus protease for 15 h. The purified protein was concentrated using Amicon Ultra centrifugal filter devices (Millipore, cut off 5 kDa).

### Denaturing and refolding protein preparation

All unfolding transitions were performed using CD spectroscopy from 25°C to 105°C, at 10-degree intervals. MTH1880 samples were incubated at each temperature for 20 min. For refolding associated with thermal denaturation, samples were denatured at 105°C and then incubated at room temperature for 24 h. All denaturant unfolding transitions were also carried out using CD spectroscopy, fluorescence spectroscopy, size exclusion chromatography, and NMR spectroscopy. In order to denature the proteins, protein samples were dialyzed against buffer containing urea or GdnHCl for 24 h. After denaturation, urea or GdnHCl was removed from the protein samples by extensive buffer exchange using dialysis for 24 h.

### Circular dichroism spectroscopy

CD spectra for ~25 μM MTH1880 were acquired in 25 mM sodium phosphate buffer (pH 8.0) using a JASCO J-815 spectrometer. Far-UV CD spectra were monitored from 200 to 250 nm. The path length was 2 mm, and instrument parameters were set to a sensitivity of ~30 millidegrees, a response time of 1 s, and a scan speed of 100 nm/min. Spectra were recorded as an average of 8 scans.

### Intrinsic fluorescence spectroscopy

Fluorescence spectra for 25 μM MTH1880 were acquired in 25 mM sodium phosphate buffer (pH 8.0) using an LS55 spectrofluorophotometer (Perkin Elmer). The sample was contained in a 1 mL temperature-controlled cuvette (25°C) and fluorescence spectra were acquired for wavelengths of 270 to 500 nm (data shown for 285 nm to 350 nm).

### Size exclusion chromatography

MTH1880 samples (1 mg in 2 ml), pre-incubated for 24 h at different urea or GdnHCl concentrations (25°C), were loaded onto a HiLoad^™^ 16/60 superdex^™^75 gel filtration column (GE Healthcare) equilibrated with sample buffer. The elution was carried out at a flow rate of 1.3 ml/min and monitored by absorbance at 280 nm. In all cases, the time that elapsed between separation and chromatography of the peaks was longer than 2 h.

### NMR spectroscopy

All NMR experiments were recorded at 36°C, using Bruker 800 and 900 MHZ spectrometers equipped with a z-shielded gradient triple resonance cryoprobe. ^1^H chemical shifts were referenced using the methyl proton signals of DSS at 0 ppm, and ^13^C and ^15^N chemical shifts were calculated indirectly using the ^1^H spectrometer frequency. Spectra were processed using NMRPipe software package [58] and analyzed using Sparky. Heteronuclear NMR experiments have been performed for resonance assignments of backbone and sidechain atoms [59]. ^15^N-HSQC spectra [60] were acquired for ^15^N-labeled protein at various concentrations of urea and GdnHCl. Chemical shift perturbations were calculated using the equation Δδ_AV_ = ((Δδ_1H_)^2^ + (Δδ_15N_/5)^2^)^1/2^, where Δδ_AV_, Δδ_1H_, and Δδ_15N_ are the average, proton, and ^15^N chemical shift changes, respectively.

### Molecular dynamics simulations

All Molecular Dynamics (MD) simulations were performed by using PMEMD.CUDA from the AMBER12 simulation package, and the ff99SB force field. Parameters for urea were included in the AMBER12 package. Simulations of solvated wild type MTH1880 (PDB: 1IQS) were performed for 500 ns per trajectory for temperatures ranging from 300 K to 525 K, in 25K intervals. Simulations of wild type MTH1880 that included urea were performed at 300 K and urea concentrations ranging from 0 to 6 M urea. The protein system was explicitly solvated with TIP3P water molecules in a rectangular box, with a 20-Å separation between the protein and edge of the water box. Periodic boundary conditions were used to minimize edge artifacts. To neutralize the system, three sodium ions were added. The particle mesh Ewald method was applied for treating long-range electrostatic interactions, and a 9.0-Å force-shifted cutoff was used for short-range non-bonded interactions. The hydrogen atoms were constrained to the equilibrium bond length using the SHAKE algorithm. In order to remove unfavorable van der Waals contacts, these systems were subjected to 1,000 steps of steepest decent minimization, followed by 1,000 steps of conjugate gradient minimization while the protein was constrained by a 500 kcal⋅mol^-1^⋅Å^-2^ harmonic potential. The entire system was then minimized by 2,000 steps of steepest decent minimization, followed by 2,000 steps of conjugate gradient minimization without harmonic restraints. The system was then subjected to 10ps of equilibration, in which the temperature was gradually raised from 30 K while using the SHAKE algorithm. After the equilibration step, the production runs were carried out for 500 ns, with a 2-fs time step, using the NPT ensemble, i.e., a constant number of particles (N), pressure (P), and temperature (T). Temperature and pressure were controlled by a Berendsen thermostat and barostat, with coupling constants of 1.0 and 2.0 ps, respectively. All trajectories were recorded every 10 ps. For each environment (temperature and urea concentration), ten independent MD simulation trajectories were performed with different initial velocities for each of which 500 ns simulation was carried out. The size of the protein was roughly estimated by radius of gyration (R_g_), which is defined as the root of the mass-weighted mean of the quadratic distances of all atoms from the center of mass. The ptraj toolset available in the AMBER12 package and in-house analysis programs were used to analyze the resulting MD trajectories. To analyze the statistics of protein secondary structure, the cpptraj of AMBER12 was used. VMD was used for the preparation of structural figures [61]. The atomic fluctuation, known as RMSF, is defined as the root of the mean squared deviation from the ensemble averaged position. The correlation matrix is defined by:$c_{i,j}=\sigma_{i,j}/\sqrt{\sigma_{i,i}\sigma_{j,j}}$, where *σ*_*i*,*j*_ is the variance-covariance matrix which is defined by:*σ*_*i*,*j*_ = 〈(***r***_***i***_ − 〈***r***_***i***_〉)⋅(***r***_***j***_ − 〈***r***_***j***_〉)〉, where ***r***_*i*_ is the three-dimensional instantaneous position vector of the *i-*th atom and〈***r***_*i*_〉 is its average position vector over the whole ensemble [62].

### Munoz-Eaton (ME) model description of the free energy landscape

. The Munoz-Eaton (ME) model [63, 64] simplifies a protein structure by a binary spin variable *m*_*i*_ for each residue *i* = 1, 2, …, N. When the dihedral angle (Φ_*i-1*_, Ψ_*i*_) for a given residue *i* is locked into that of a native state, the value 1 is assigned to a binary spin variable *m*_*i*_. Otherwise, *m*_*i*_ takes the value 0. Therefore, the sequence of one-dimensional binary numbers (1 or 0) approximates the ensemble structures of a protein. The conformational free energy of a protein (per mol) in the original ME model, where the pairwise-interaction energy between two residues residing within the cutoff distance was taken as -1, or otherwise 0, by the amino acids’ sequence-independent manner, could be extended to include the amino acids’ sequence-dependent interaction energies as follows:

$$\Delta \text{G}\left(\left\{m_{k}\right\}\right)=\underset{\begin{array}{c} \langle i,j\rangle \\ j\geq i+3 \end{array}}{\sum }\left(E_{ij}^{coulomb}+E_{ij}^{vdW}+E_{ij}^{pol.sol.}+E_{ij}^{non-pol.sol.}\right)\overset{j}{\underset{k=i}{\prod }}m_{k}-T\overset{N}{\underset{k=1}{\sum }}\Delta S_{k}\cdot m_{k}$$

A nonzero value of the product *m*_*i*_ ⋅ *m*_*i+1*_ ⋅ ⋅ ⋅ ⋅ ⋅ *m*_*j*_ implies that all peptide bonds between the residue *i* and *j* are set with the native-dihedral angles, which then allows the establishment of native interactions in the configuration between the residues *i* and *j*. *T* denotes the temperature. The pairwise-interaction energy between residues *i* and *j* is calculated by summing up all pairwise-interaction energies between atoms belonging to residue *i* and atoms belonging to residue *j*. Therefore, one can include the heterogeneous nature of these pairwise-interaction energies at the atomic level, which then leads to the sequence dependent pairwise-interaction energies at the amino acid level. These pairwise-interaction energies consist of electrostatic (Coulomb) energy *E*_*ij*_^*coulomb*^, van der Waals energy *E*_*ij*_^*vdW*^, and polar solvation energy *E*_*ij*_^*pol*.*sol*.^. The values of these interaction energies between pairs of atoms were obtained from the atomistic information gained from MD simulations. E_ij_^non-pol.sol.^ was(-7.2 cal⋅mol^-1^Å^-2^)×A_ij_, where A_ij_ is the sum of solvent accessible surface areas of nonpolar atoms of the *i*^th^ residue covered by all (polar + nonpolar) atoms of *j*^th^ residue. Given the amino acids’ sequence and the equilibrium structural ensembles from MD simulations, AMBER and in-house analysis tools can readily provide all the necessary information for for extracting *E*_*ij*_^*coulomb*^, *E*_*ij*_^*vdW*^, *E*_*ij*_^*pol*.*sol*.^, *and E*_*ij*_^*non-pol*.*sol*.^. The conformational entropy reduction *ΔS*_*k*_ of residue *k* is set as -3.8 kcal/mol⋅K if the secondary structure of the residue *k* is a *helix or sheet* and at -1.3 kcal/mol⋅K if the secondary structure of the residue *k* is a *turn or loop*. The Boltzmann weight contribution ω({*m*_*k*_}) = exp[−Δ*G*({*m*_*k*_})/*RT*] of any configuration *{m*_*k*_*} = (m*_*1*_, *m*_*2*_, *…*, *m*_*N*_*)* to the partition function Z = Σ_{mk}_*ω*({*m*_*k*_}) is the product of the weights of the stretches of native binary variables contained in that configuration; $\omega_{j,i}=\text{exp}\left[-\frac{1}{RT}\left(\sum_{k=i}^{j-1}\sum_{l=k+1}^{j}\left\{E_{kl}^{coulomb}+E_{kl}^{vdW}+E_{kl}^{pol.sol.}+E_{kl}^{non-pol.sol.}\right\}-T\sum_{k=i}^{j}\Delta S_{k}\right)\right]$ for a native stretch going from residue *i* to residue *j* (*i*+3≤*j*). One could rewrite the partition function by Z = ∑_*q*_*Z*_*q*_ where *q* is the number of native residue and *Z*_*q*_ is a restricted partition function. Now, the free energy as a function of the number (or fraction) of native residue is given as ΔG(q) = −*RT* log *Z*(*q*).

## Results and Discussion

### Temperature-induced unfolding of MTH1880 is consistent with a two-state mechanism

**Fig 1:**
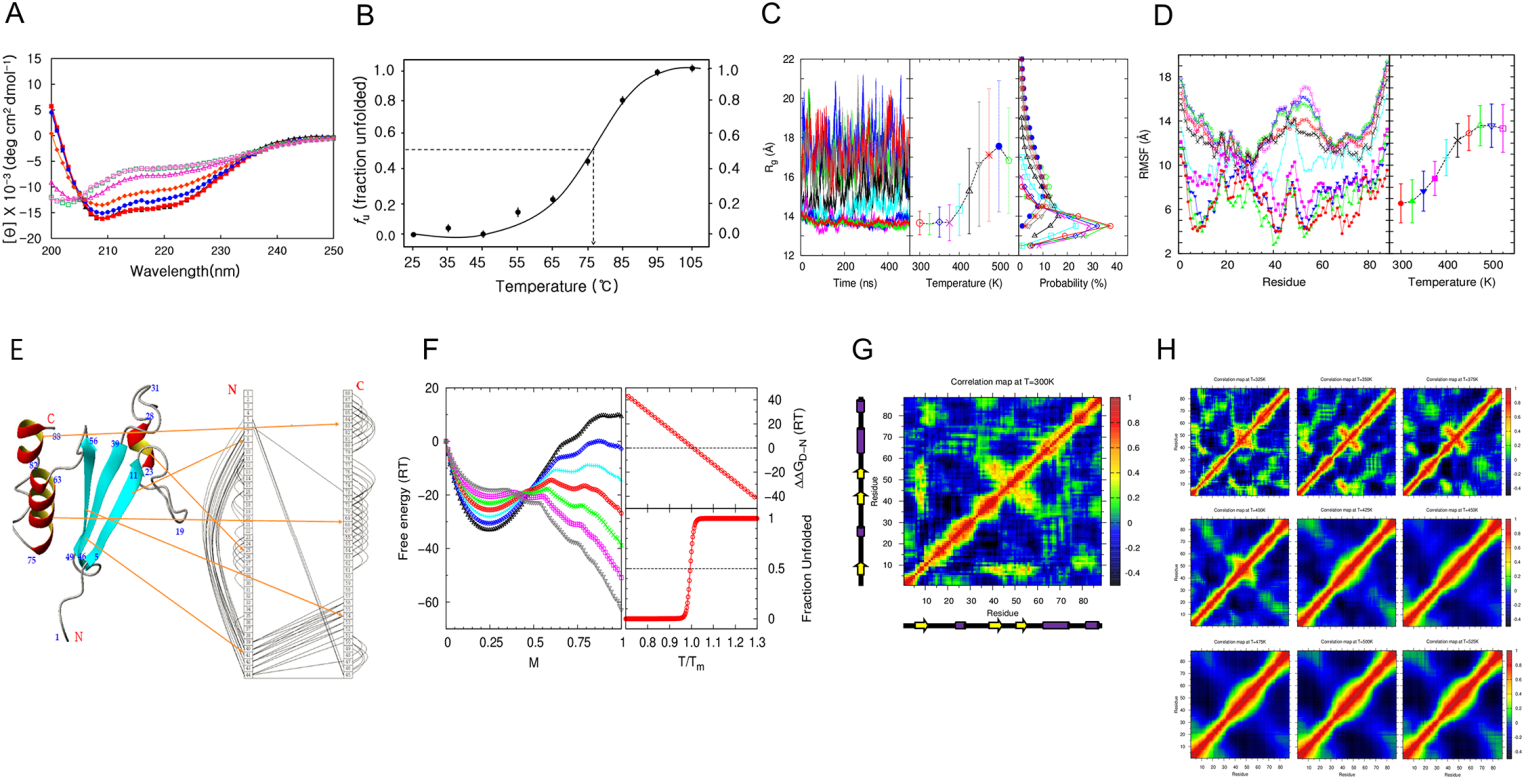
Thermal folding-unfolding of MTH1880. (A) Data were acquired at 25°C (black triangle), 45°C (red square), 65°C (blue circle), 75°C (yellow square), 85°C (red triangle), 95°C (green square), and 105°C (red circle). Protein concentration was ~25 μM in a cell of 0.1 mm path length. (B) The fraction of unfolding extracted from far-UV CD spectra at 222 nm with a constant heating rate of 10°C/h as a function of temperature was plotted and fit by a sigmoidal curve. The transition mid-temperature (T_m_) of MTH1880 was 76 ± 0.5°C. (C) Results from molecular dynamics (MD) simulations. (Left panel) Radius of gyrations (R_g_) of MTH1880 as a function of time at temperatures from 300 K to 525 K. (Middle panel) R_g_ averaged over 400,000 snapshots in the time window from 100 ns to 500 ns. The error bar denotes one standard deviation. (Right panel) The probability distribution of R_g_ is plotted for each temperature and results in sharp distributions at low temperatures and broad distributions at high temperatures. (D) (Left panel) Atomic fluctuation (RMSF) of MTH1880 by residue in the same time window and same temperatures as (C). (Right panel) RMSF averaged over 88 residues. (C, D) This data were acquired at 300K(red filled circle), 325K(green filled triangle), 350K(blue filled triangle), 375K(pink filled square), 400K(cyan cross), 425K(black cross), 450K(red circle), 475K(green triangle), 500K(blue triangle), and 525K(pink square). (E) Secondary structures of MTH1880 are shown with residue numbers. A ladder diagram displays residue-residue pairwise contacts denoted by a semicircle line with the distance cut-off 6.5A to emphasize the major topology of pairwise residue-residue interactions based on three-dimensional structure of MTH1880. (F) Extended Munoz-Eaton (ME) model. Data were acquired at 0.7T_m_(black reverse triangle), 0.8T_m_(pink square), 0.9T_m_(green cross), 1.0T_m_(red circle), 1.1T_m_(cyan cross), 1.2T_m_(blue square), and 1.3T_m_(black triangle). (Left panel) Free energy (ΔG) landscape of MTH1880 as a function of the reaction coordinate, where M = 0 denotes the fully denatured structure and M = 1 denotes the native structure. The free energy of folding ΔΔG_D-N_ (ΔG_D_—ΔG_N_, right top panel) and the fraction of unfolded protein as a function of the reduced temperature (T/T_m_), where T_m_ is the transition mid-temperature (right bottom panel). At T = T_m_, ΔΔG_D-N_ = 0, and the fraction of unfolding is 0.5. (G) Correlation matrix of MTH1880 at 300 K is calculated from 400,000 snapshots in the same time window as (C). The secondary structure of MTH1880, in the N to C direction, are depicted next to the axes. (H) Correlation matrix of MTH1880 at (Top left to right) 325K, 350K, 375K, (Middle left to right) 400K, 425K, 450K, (Bottom left to right) 475K, 500K and 525K.

To investigate the thermodynamic stability of MTH1880 and its folding-unfolding mechanism, thermal unfolding experiments were performed and monitored by circular dichroism (CD) spectroscopy. Far-UV CD spectra for MTH1880, from 25°C to 105°C, at 10-degree intervals, are shown in Fig 1A. A strong α-helical signal persists at 25°C, 35°C, and 45°C, implying that MTH1880 contains a substantial amount of secondary structure and remains in a native fold in this temperature range. The α-helical signal begins to diminish at 55°C, disappears rapidly between 75°C and 85°C, and is completely denatured at 95°C, with a final α-helix content of 5.25%. Our results imply that MTH1880 undergoes a large conformational change as the temperature changes from 25°C to 105°C. The fraction of unfolded protein (*f*_U_), as a function of temperature, could be fit by a sigmoidal curve (Fig 1B); the melting temperature (T_m_) of MTH1880, defined at *f*_U_ = 0.5, is T_m_ = 76 ± 0.5°C. The thermodynamic stability of MTH1880 is high and unfolding with thermal denaturation is consistent with a two-state transition. We examined the effect of Ca^2+^ on the thermal stability of MTH1880. Thermal denaturation of MTH1880 containing Ca^2+^ were measured using the Far-UV experiments from 25°C to 105°C, at 10-degree intervals (Figs A and B in S1 File). The fraction of unfolded protein (*f*_U_), as a function of temperature in the presence of Ca^2+^ is fit to a sigmoidal curve (Figs A and B in S1 File, in red line); the melting temperature (T_m_) of MTH1880 in the presence of Ca^2+^ is determined as 75.8 ± 0.5°C. This suggests that the existence of Ca^2+^ does not affect thermal stability. Therefore, we conclude that Ca^2+^ binding is related to molecular function of MTH1880 as a Ca^2+^buffering protein, not to thermo-stability.

The structural dynamics of MTH1880 were simulated by molecular dynamics (MD) simulation using the AMBER12 ff99SB force field at several temperatures in the pre- and post-transition regions. The average radius of gyration (R_g_) and RMSF for MTH1880, simulated for temperatures ranging from 300K to 525K, at 25K intervals, is shown in Fig 1C and 1D. The left (left and right) panel of Fig 1C and 1D shows the values of R_g_ (RMSF) averaged over 10 different trajectories for different temperatures. The distribution of R_g_ values from 400,000 equilibrated conformations for each temperature demonstrates that MTH1880 undergoes a structural transition from native conformations at low temperatures, with a narrow R_g_ distribution centered at 14 Å, to denatured conformations at high temperatures, with a very broad R_g_ distribution (middle panel of Fig 1C). The melting temperature T_m_ is approximately 425K, which separates the native states from denatured states. In order to provide qualitative and systematic insight into the nature of the folding-unfolding mechanism of MTH1880, we employed the extended Munoz-Eaton (ME) model to construct the free energy landscape. Based on the structural information obtained for native MTH1880 from MD simulations, we not only constructed a pairwise map with a distance cut-off 6.5A between C_α_’s of two amino acids to identify the major topology of residue-residue interactions (Fig 1E) but also calculated their pairwise interaction energies after coarse-graining the pairwise interaction energies between all atoms using the AMBER12 ff99SB force field. This was used as an energy function in the extended Munoz-Eaton (ME) model [57] used to construct the free energy landscape. The left panel of Fig 1F shows the free energy landscape from 0.7T_m_ (gray curve) to 1.3T_m_ (black curve) as a function of the fraction (M) of native residues. With continuous temperature change, the position of the global minimum of the free energy landscape moves discontinuously from M = 1.0, which corresponds to native states at low temperatures, to M = 0.25, which corresponds to denatured states at high temperatures. The free energy of folding (ΔΔG_D-N_) as a function of temperature suggests that the free energy values for the native and denatured states are the same at T_m_ (top right panel of Fig 1F) and that the height of the free energy barrier is about 11RT_m_, implying that MTH1880 is not only a highly stable protein, but also undergoes a two-state folding transition without the presence of meta-stable intermediates (red curve of left panel of Fig 1F). Consistent with our CD measurements, the fraction of unfolding as a function of reduced temperature (T/T_m_), based on the extended Munoz-Eaton (ME) model (57), predicts that unfolding occurs through a two-state transition without an intermediate state (bottom right panel of Fig 1F). The correlation (normalized variance-covariance) matrix of MTH1880 at 300 K is calculated from 400,000 snapshots in the same time window as Fig 1C and is shown in Fig 1G. The secondary structures of MTH1880, from N to C direction, are depicted next to the axes. It demonstrates that the interactions among β1, β2, and β3 are strongly intact which provides the native structure of MTH1880 with the structural stability, The same correlation matrix of MTH1880 at temperatures ranging from 300K to 525K, at 25K intervals are also presented in Fig 1H, which show the disappearance of these interactions among β1, β2, and β3 as the temperature rises above the folding mid-temperature.

### Urea-induced unfolding of MTH1880

**Fig 2:**
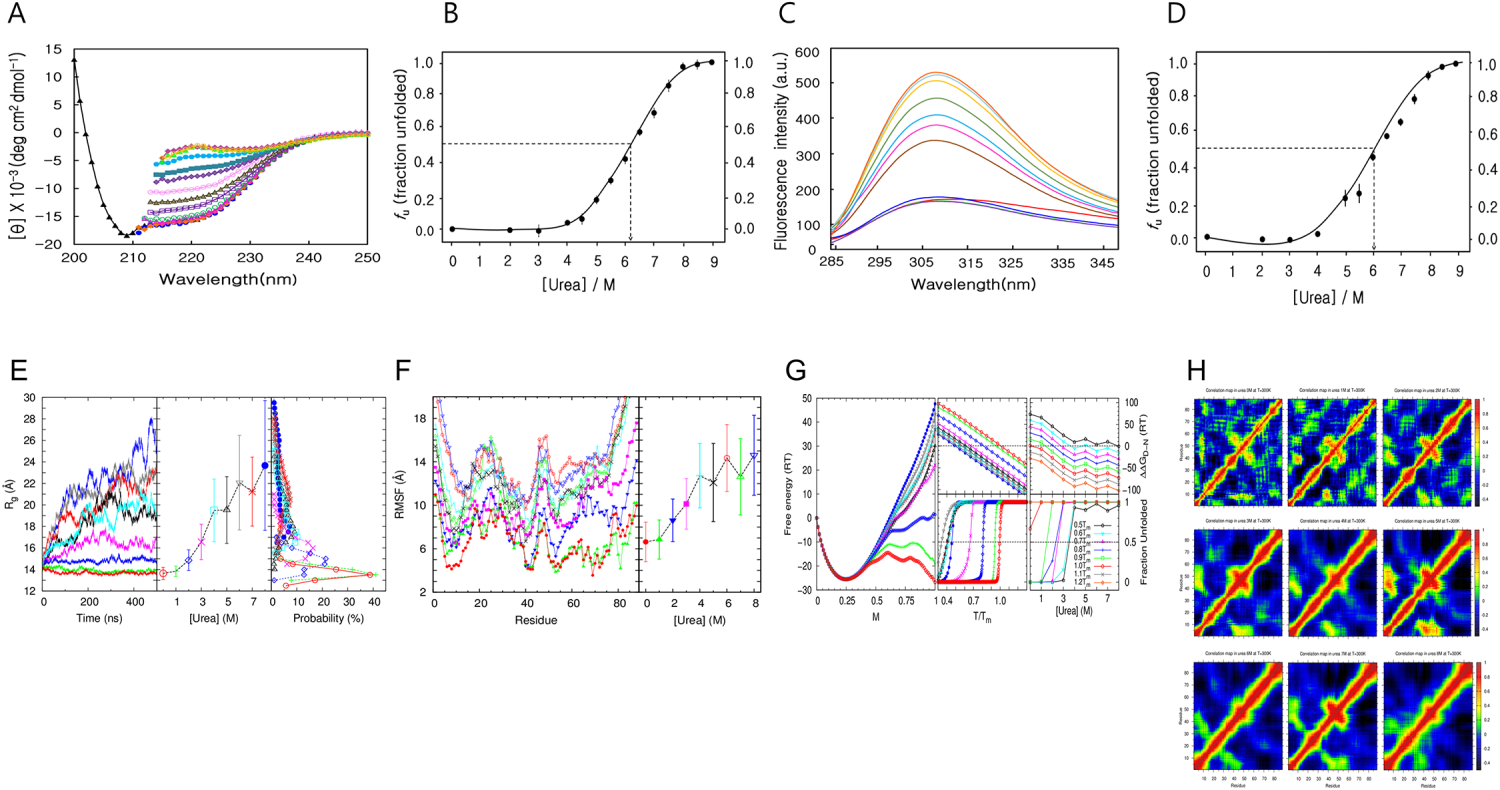
Unfolding of MTH1880 in the presence of urea. (A) Data were acquired at 0 M (black triangle), 2 M (blue circle), 3 M (yellow diamond), (red square), 4.5 M (green circle), 5 M (magenta square), 5.5 M (dark green triangle), 6 M (red circle), 6.5 M (magenta filled diamond), 7 M (green square), 7.5 M (green triangle), 8 M (blue triangle), 8.5 M (khaki diamond) and 9 M (yellow circle) urea. (B) The fraction of unfolded protein extracted from far-UV CD spectra (25 μM) at 222 nm as a function of urea concentration was plotted and fit by a sigmoidal curve. The transition mid-concentration of urea (C_m_) is 6.10 ± 0.15 M. (C) Fluorescence spectra acquired for urea concentrations ranging from 0 to 9.0 M. Data were acquired for 0 M (red line), 2M (fluorescent green line), 3 M (purple line), 4 M (blue), 6 M (brown), 6.5 M (magenta), 7 M (sky-blue), 7.5 M (khaki), 8 M (yellow), 8.5 M (light sky-blue) and 9 M (orange). (D) The fraction of unfolded protein extracted from fluorescence-emission spectra (25 μM) at 308 nm as a function of urea concentration was plotted and fit by a sigmoidal curve. The transition mid-concentration of urea is 6.00± 0.05 M. (E) Results from MD simulations. (Left panel) R_g_ of MTH1880 as a function of time, with urea concentrations ranging from 0 to 8 M at 300 K. (Middle panel) R_g_ averaged over 300,000 snapshots taken in the time window of 200 to 500 ns. The error bar denotes one standard deviation. (Right panel) The probability distribution of R_g_ is plotted for each urea concentration, and results in sharp distributions at low temperatures and broad distributions at high temperatures. (F) (Left panel) RMSF of MTH1880 by residue in the same time window, same temperatures and same urea concentration as (E). (Right panel) RMSF averaged over 88 residues. (E, F) This data were acquired for urea 0M(red filled circle), 1M(green filled triangle), 2M(blue filled triangle), 3M(red square), 4M(cyan cross), 5M(black cross), 6M(red circle), 7M(green triangle), and 8M(blue triangle). (G) Results from employing the extend ME model. Data were acquired for urea 0M(red circle), 1M(green cross), 2M(blue square), 3M(red cross), 4M(cyan square), 5M(black triangle), 6M(grey triangle), 7M(purple star), and 8M(blue filled circle). (Left panel) Free energy landscape of MTH1880 at 1.0T_m_ for each urea concentration as a function of M. (Middle top panel). The free energy of folding ΔΔG_D-N_ (middle bottom panel) and the fraction of unfolded protein for each urea concentration, as a function of T/T_m_. At T = T_m_, ΔΔG_D-N_ = 0, and the fraction of unfolding is 0.5. (Right top panel) The free energy of folding ΔΔG_D-N_ (right bottom panel) and the fraction of unfolded protein for the temperature interval of 0.1T_m_ to 1.2T_m_, as a function of urea concentration. At the transition mid-concentration of urea, ΔΔG_D-N_ = 0, and the fraction of unfolding is 0.5. (H) Correlation matrix of MTH1880 is calculated from 300,000 snapshots in the same time window as (E) with urea concentration (Top left to right) 0M, 1M, 2M, (Middle left to right) 3M, 4M, 5M, (Bottom left to right) 6M, 7M and 8M.

Far-UV CD spectra of MTH1880 at 13 different urea concentrations (2, 3, 4, 4.5, 5, 5.5, 6, 6.5, 7, 7.5, 8, 8.5, 9 M) are presented in Fig 2A. The molar ellipticity remains constant for urea concentrations of 2 M, 3 M and 4 M, but starts to degrade from 4.5 M, and disappears above 8M, implying that MTH1880 denatures completely. Although full secondary structural analysis for wavelengths < 210nm is not shown because the UV absorption is disturbed by urea, it is still possible to estimate the fraction of helix from the molar ellipticity at 222 nm. Fig 2B shows the fraction unfolded (*f*_U_), calculated from the molar ellipticity of MTH1880 at 222 nm, as a function of urea concentration. No obvious change in the ellipticity is observed when the urea concentration is below 4 M, whereas a molecular transition that reflects a disruption of secondary structure occurs above 4 M. A sigmoidal fit suggests that the mid-concentration C_m_ of MTH1880, defined at *f*_U_ = 0.5, is 6 .1± 0.15 M, again demonstrating that the unfolding of MTH1880 by urea denaturation is consistent with a two-state transition.

The intrinsic fluorescence spectrum for MTH1880 is shown in Fig 2C. MTH1880 has two tyrosine residues (Tyr25 and Tyr59) and several phenylalanine residues, but no tryptophan. In its native state, MTH1880 exhibits an emission spectrum with a maximum value at 311 nm (excitation 275 nm). Increasing the urea concentration from 2 M to 9 M caused a red-shift in λ_max_ in the emission spectrum from 311 nm to 308 nm. Fluorescence intensity was constant for 2 M, 3 M, and 4 M urea, and reached a maximum at 9 M urea, indicating that MTH1880 fully unfolded (Fig 2C). The C_m_ for equilibrium unfolding, calculated for 308 nm, is 6 ± 0.1 M, which indicates that the urea-induced unfolding of MTH1880 is a two-step process (Fig 2D).

Conformational snapshots of MTH1880 MD simulations performed at 300 K, with urea concentrations ranging from 0 to 8 M, at 1-M intervals, were collected for structural analysis. The left panel of Fig 2E shows the values of R_g_ averaged over 10 different trajectories from MD simulations at different urea concentrations. The value of R_g_ averaged over 300,000 equilibrated conformations (the distribution of these R_g_ values) for each urea concentration tested is presented in the middle (right) panel of Fig 2E. Below 4 M, the R_g_ value equilibrated after 200 ns, whereas above 5 M, even 500 ns was insufficient time to equilibrate MTH1880 conformation, meaning that the structure was not yet unfolded. However, we expected that MD simulations for urea concentrations above 5 M, and acquired on a longer time scale, would produce MTH1880 conformations with larger R_g_ values, reflecting an ensemble of denatured states. These results suggest that MTH1880 undergoes a structural transition from native conformations at low urea concentrations, with a narrow R_g_ distribution, to denatured conformations at high urea concentrations, with a very broad R_g_ distribution. The similar results were drawn also from the analysis of RMSF as presented in Fig 2F. The free energy landscape as a function of the fraction of native residues, from 0 to 8 M urea and 0.5 T_m_ to 1.2 T_m_ (T_m_ is the melting temperature at 0 M urea), was calculated by employing the extended ME model [57]. The free energy landscape for T = 1.0T_m_ suggests that as urea concentration changes continuously, the global minimum of the free energy landscape moves discontinuously from M = 1.0, which corresponds to native states at low urea concentrations, to M = 0.25, which corresponds to denatured states at high urea concentrations (left panel of Fig 2G). The free energy of folding (ΔΔG_D-N_) and the fraction unfolded as a function of temperature (urea concentration) for different urea concentrations (temperatures) suggests that the value of T_m_ decreases (C_m_ increases) as the urea concentration increases (temperature decreases) (middle panel of Fig 2G). Together, these results indicate that MTH1880 undergoes a two-state transition with urea denaturation, without a meta-stable intermediate state. The results from the analysis of the correlation matrix, presented in Fig 2H, show the persistence of these interactions among β1, β2, and β3 as the urea concentration rises until 8M urea concentration despite above the folding mid-concentration of urea.

### GdnHCl-induced unfolding

**Fig 3:**
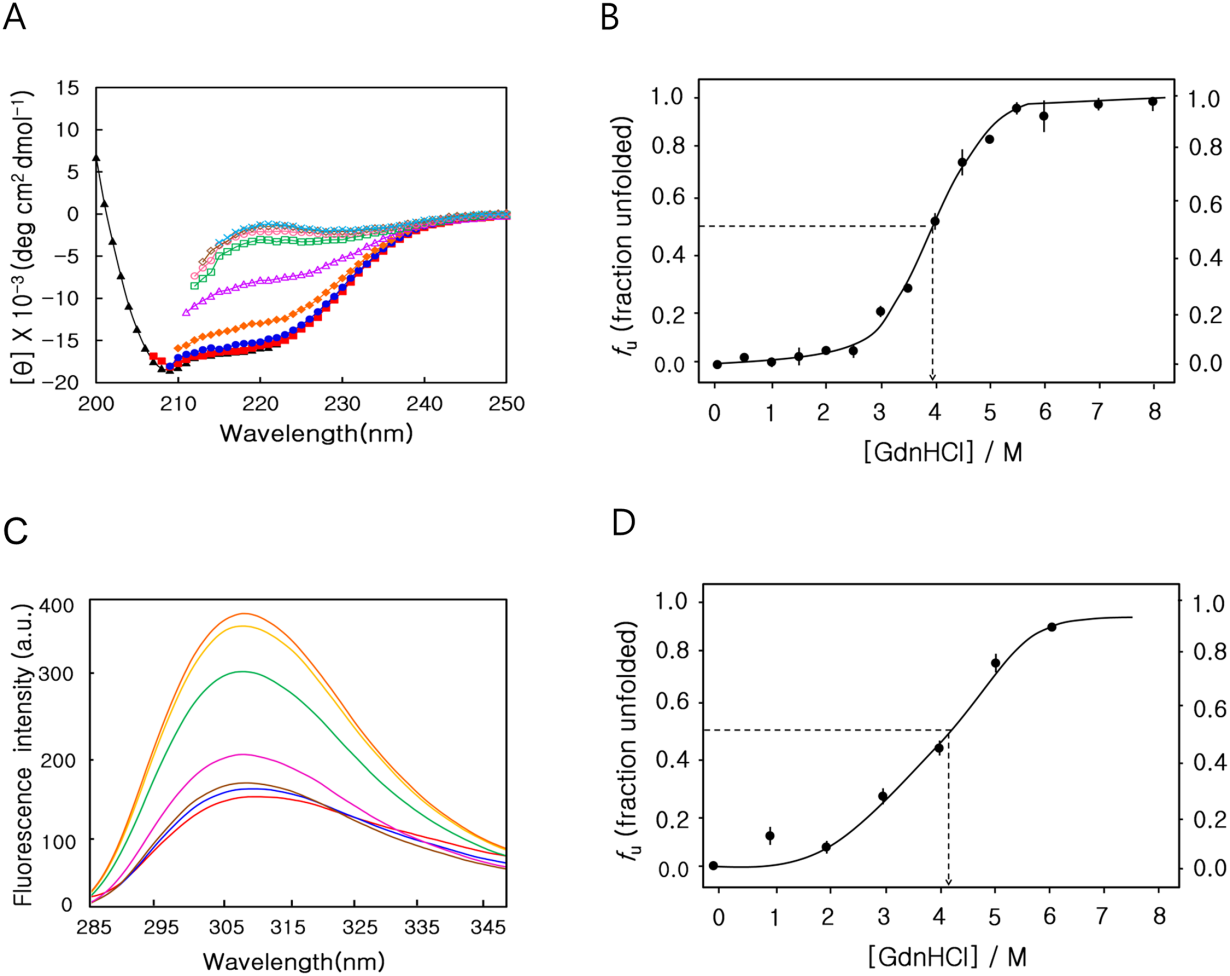
GdnHCl denaturation monitored by circular dichroism and fluorescence spectroscopy. (A) Data were acquired at 0 M (black triangle), 1 M (red square), 2 M (blue circle), 3 M (hkaki filled diamond),4.5 M (red triangle), 5 M (green square), 6 M (red circle), 7 M (hkaki diamond), and 8 M (cyan cross) urea. (B) The fraction of unfolding extracted from far-UV CD spectra (25 μM) at 222nm as a function of GdnHCl concentration was plotted and fit by a sigmoidal curve. The transition mid-concentration of GdnHCl (C_m_) is 3.95 ± 0.1 M. (C) Fluorescence-emission spectra for different GdnHCl concentrations ranging from 0 to 6.0 M. (D) The fraction of unfolding extracted from fluorescence-emission spectra (25 μM) at 308 nm, as a function of urea concentration, was plotted and fit by a sigmoidal curve. The transition mid-concentration of urea is 4.1 ± 0.05 M.

We denatured MTH1880 by GdnHCl, a stronger denaturant than urea, in order to induce the complete unfolding of MTH1880 (22–23). Far-UV CD spectra for MTH1880 were acquired for 14 different GdnHCl concentrations ranging from 0.5 to 8 M (Fig 3A). The molecular ellipticity and the fraction unfolded were nearly unchanged for GdnHCl concentrations up to 2.5 M, but disappeared above 6 M (Fig 3A and 3B). A sigmoidal fit to the unfolding curve indicates that C_m_ is 3.95 ± 0.1 M. Native fluorescence at 308 nm was used to monitor unfolding in the presence of 0 to 6 M GdnHCl (Fig 3C and 3D); consistent with thermal and urea denaturation, MTH1880 unfolding due to GdnHCl denaturation is consistent with a two-state transition mechanism.

### Reversible unfolding of MTH1880

**Fig 4:**
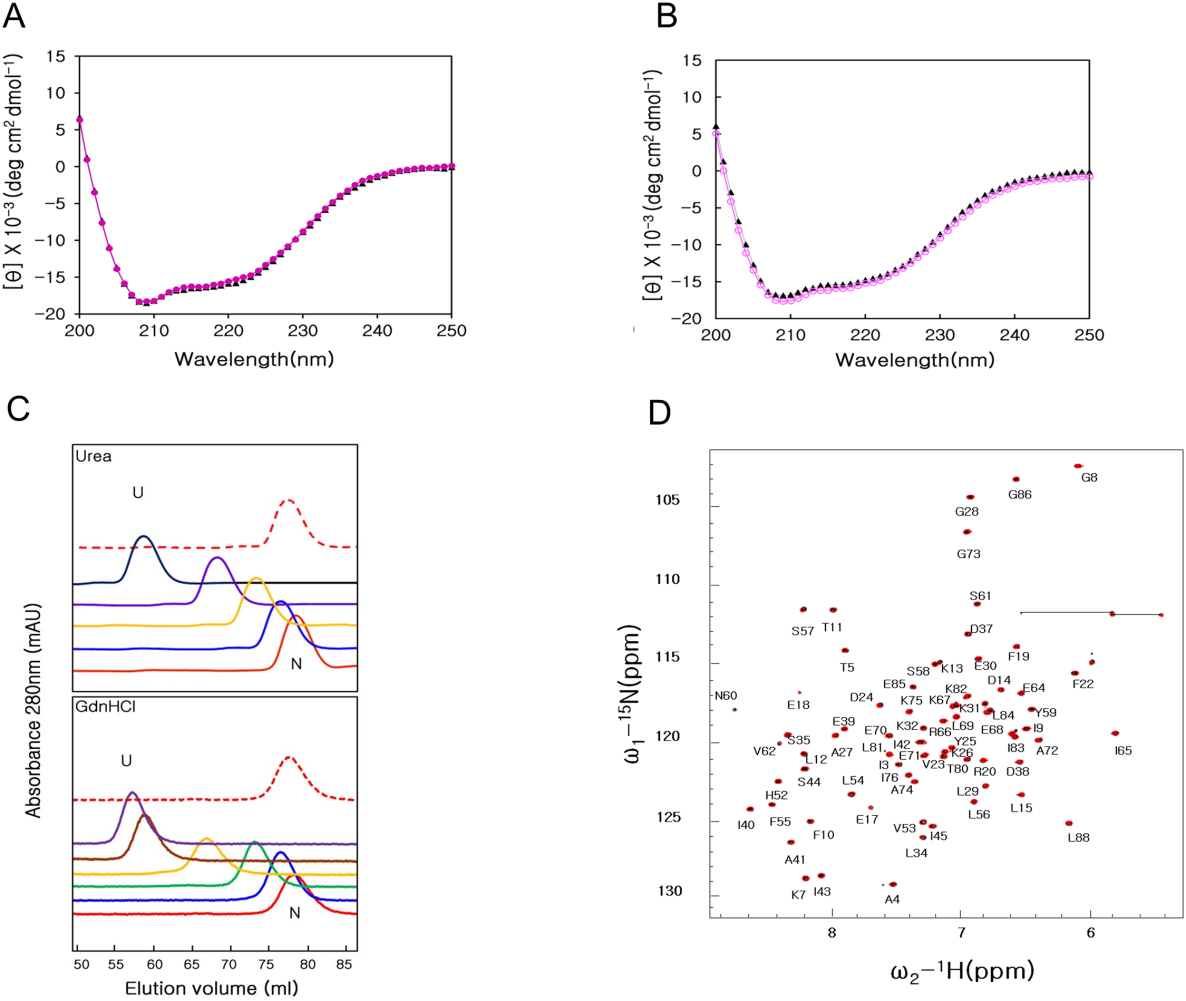
Refolding process of MTH1880. (A) Superimposition of Far-UV CD spectra for the native state (black triangle) and refolded state (red circle), which was reached by lowering to 25°C from 105°C. (B) Far-UV CD spectra for the native state (black triangle) and refolded state (red circle), which was reached by diluting GdnHCl from 6 to 0 M. (C) Size-exclusion FPLC profiles of MTH1880 as a function of urea and GdnHCl concentration of 0 M (red line), 2 M (blue line), 3 M (green line), 4 M (yellow line), 5 M (brown line), 6 M (purple line), 8 M (black line), and 0 M (refolded, red dotted line). (D) Overlay of the two-dimensional ^1^H-^15^N HSQC spectra for MTH1880 are shown in both the native state (black) and refolded state (red) at 36°C.

We examined whether thermal or GdnHCl denaturation of MTH1880 was reversible. Far-UV CD spectra for MTH1880 were acquired after the complete denaturation of MTH1880 at 105°C for 1 h, followed by incubation at 25°C for 24 h. The secondary structure of the refolded state, as measured by CD, is nearly identical to that of the native state (Fig 4A). MTH1880 structure after GdnHCl denaturation and refolding was also examined by CD (Fig 4B). After completely unfolding MTH1880 using 6 M GdnHCl for 24 h, the sample was extensively dialyzed to remove GdnHCl. Size exclusion FPLC profiles for MTH1880 as a function of urea and GdnHCl concentration are shown in Fig 4C. MTH1880 was incubated with increasing concentrations of urea or GdnHCl for 24 h at 25°C, and then applied to a size exclusion column equilibrated with urea or GdnHCl. The elution volume of the native state is normally larger than that of unfolded state. If the exchange between the two states occurs on the same time scale as the chromatographic separation, both states can be isolated an individual elution peaks. Refolded MTH1880 elutes at 78 ml, which is the same as the native state (Fig 4C, red dotted line). The reversibility of the unfolding reaction was also confirmed by NMR spectroscopy. All resonances of MTH1880 are identical in the two-dimensional ^15^N-^1^H HSQC for the native and refolded states (Fig 4D), indicating that the unfolding-refolding transition associated with thermal or GdnHCl denaturation is completely reversible.

### Structural basis of hyper-stability of MTH1880

**Fig 5:**
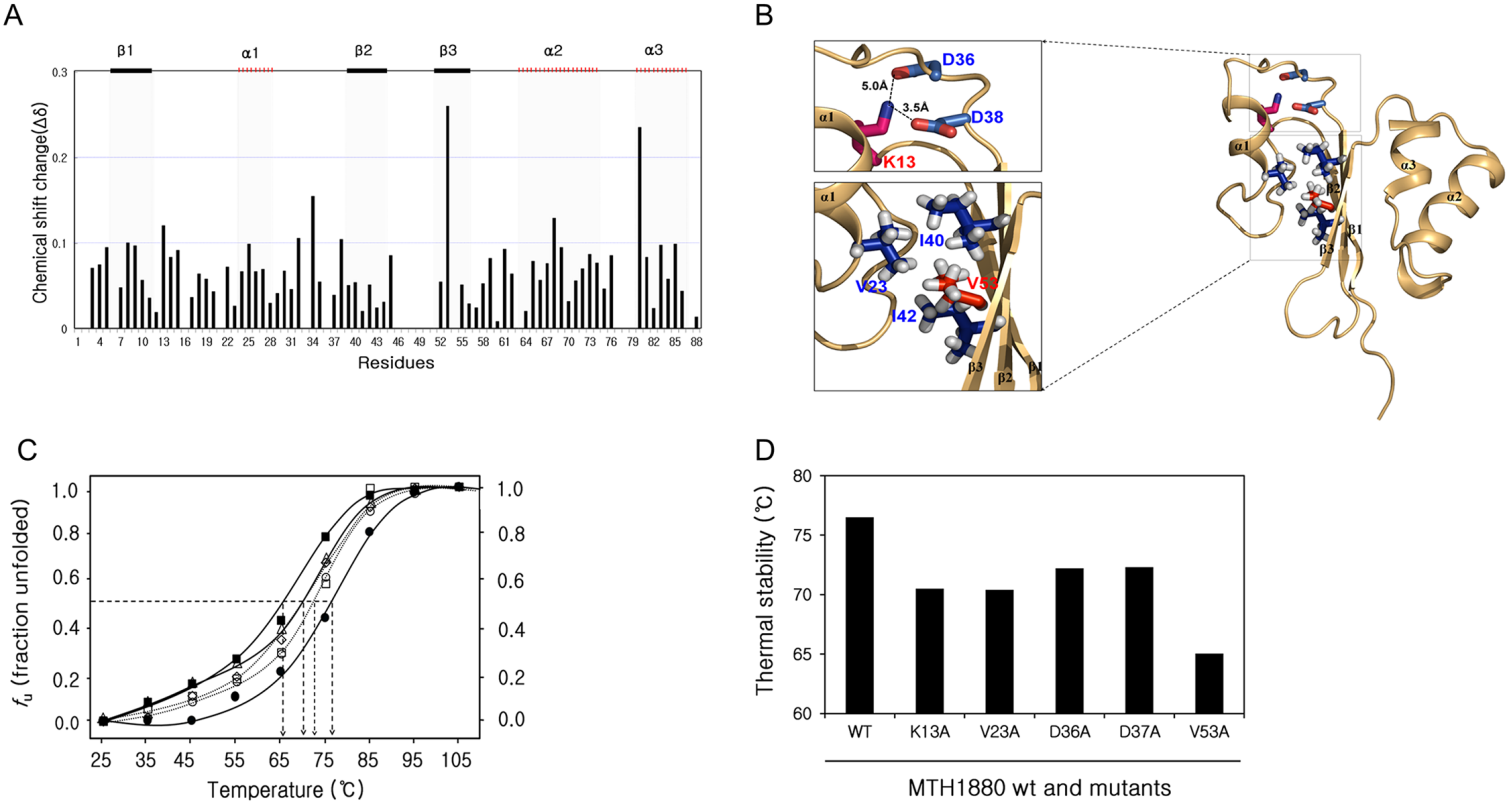
Residue-specific characteristics of MTH1880 unfolding. (A) Chemical shift perturbation (CSP) analysis to detect residues susceptible to urea denaturation. The average chemical-shift changes were calculated using the following formula: Δδ_AV_ = [(Δδ_1H_)^2^+(Δδ_15N_/5)^2^]^1/2^, where Δ*δ*_AV_, Δ*δ*_1H_, and Δ*δ*_15N_ are the average, proton, and ^15^N chemical-shift changes, respectively. (B) Structure of the MTH1880 represented by a ribbon diagram. K13-D36 and K13-D38 form salt bridges. Dashed lines indicate the salt bridges. K13-D36 and K13-D38 salt bridges contributed to the stability of the folding structure of MTH1880. Red and blue atoms mean oxygen and nitrogen, respectively. Hydrophobic core is formed by side-chain connectivity of hydrophobic residues. It is represented by sphere and stick, respectively. (C) Thermal-induced denaturation curves of wild type MTH1880 and mutants. The fraction of unfolding extracted from far-UV CD spectra at 222 nm with a constant heating rate of 10°C/h and 25μM protein concentrations. MTH1880 wt (filled circle), mutants in the salt bridge; K13A (triangle), D36A (square), D38A (circle) and mutants in the hydrophobic pocket; V23A (diamond), V53A(filled square). (D) Thermal stabilities were investigated for MTH1880 mutants. Tm values of mutant proteins are indicated as black bars.

MTH1880 consists of three α-helices and three anti-parallel β-strands. Hydrophobic interactions are the main forces that stabilize the structure of the MTH1880 and the electrostatics interactions also contribute to the structural integrity. The hydrophobic cores of the folded MTH1880 are constituted by residues; Phe10/Leu15/Val23/ Ile40/ Ile42, Leu15/Pro16/ Pro21/ Val23/ Leu40, Val23/Tyr25/ Ile40/ Ile42/ Val53, Val53/Val23 /Ile40/ Ile42, Leu54/Leu69/ Ala72/ Leu84/ Leu88, Leu56/Ala41/ Tyr59/ Leu69, Leu84/Leu54/Leu81/ Ile83. Salt bridges responsible for protein stability are Lys7-Glu70, Lys13-Asp36, Lys13- Asp38, Asp63-Arg66, Arg66-Glu70, Lys67-Glu71, Glu68-His87 and Glu71-Lys75. Based on the unfolding curve, 2D HSQC experiment was carried out for MTH1880 at 6 M urea condition. A series of HSQC experiments were performed for 2 M, 3 M, 4 M, and 5 M urea environments. Several residues (Lys13, Leu34, Val53 and Lys68) experienced large chemical shift perturbations (Fig 5A). Previous folding studies for thermophilic proteins proposed that both salt bridge and hydrophobic interactions could play an important role in thermostability [34–36]. Three-dimensional structure of MTH1880 provided seven residues which are responsible for stability of protein via both electrostatic and hydrophobic interactions (Fig 5B). To identify residues responsible for hyper-stability of the MTH1880, we chose seven residues that mainly involved in salt bridge (Lys13, Asp36 and Asp38) and hydrophobic interactions (Val23, Ile40, Ile42 and Val53). All residues are mutated to Ala, retaining stability of structure involved in hydrophobic interaction. Five mutants (Lys13, Asp36, Asp38, Val23 and Val53) exhibited sigmoidal melting curves, indicative of cooperative thermal unfolding (Fig 5C). Two mutants (Ile40 and Ile42) are aggregated after protein expression, indicating that two residues are involved in structural integrity. All mutant proteins had lower thermal unfolding temperatures than that of wild-type (Figs C, D, E, F and G in S1 File). T_m_ values of the mutants are 70°C for K13A, 72.5℃ for D36A and D38A, 70°C for D23A and 65°C for V53A, respectively (Fig 5D). Lys13 mutation becomes unstable, breaking a salt bridge with Asp36 and Asp38 (Fig 5B). Especially, Val53 located in β3 involves in hydrophobic core with Val23, Ile40 and Ile42 and it plays a critical role in maintaining protein thermal stability of MTH1880.

## Conclusion

We found that (a) MTH1880 undergoes a reversible folding-unfolding process induced by urea, GdnHCl and temperature; (b) unfolding curves measured in the presence of urea or GdnHCl represented a two-state transition model; (c) β3-strand located in the middle of MTH1880 kept the native structure against the unfolding factors; and (d) urea denatured MTH1880 through a direct mechanism. We conclude that data from spectroscopic measurements, in combination with computational simulations clarify the thermodynamic and structural basis for the hyper-stability of thermophilic MTH1880 proteins.

## Supporting Information

S1 File Far-UV CD spectra of MTH1880 and mutants. **(A)** Far-UV CD spectra of MTH1880 in the presence of Ca^2+^. Data were acquired at 25°C (black triangle), 45°C (red square), 65°C (blue circle), 75°C (hkaki diamond), 85°C (red triangle), 95°C (green square), and 105°C (red circle). Protein concentration was ~25 μM in a cell of 0.1 mm path length. **(B)** The fraction of unfolding extracted from far-UV CD spectra at 222 nm with a constant heating rate of 10°C/h as a function of temperature in the presence of Ca^2+^(red filled circle) and the absence of Ca^2+^ (black filled circle) are shown by a sigmoidal curve. The transition mid-temperature (T_m_) of MTH1880 in the presence of Ca^2+^ was 75.8 ± 0.5°C. Far-UV CD spectra of MTH1880 mutants in the salt bridge and hydrophobic pocket at temperature values ranging from 25°C to 95°C **(C-G).** Data were shown at 25°C (**black** line), 45°C (red line), 65°C (blue line), 75°C (purple line), 85°C (pink line) and 95°C (green line), respectively.
(PDF)
