# Supplementary figures and images for: Probing the Folding-Unfolding Transition of a Thermophilic Protein, MTH1880
Source: PLoS One. 2016 Jan 14;11(1):e0145853. doi: 10.1371/journal.pone.0145853 (PMC4713090; doi:10.1371/journal.pone.0145853)

A

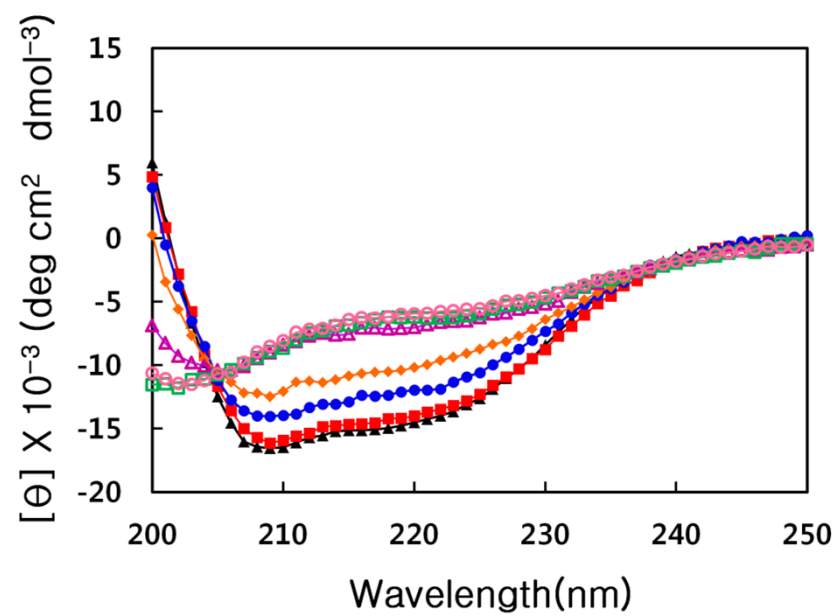

B

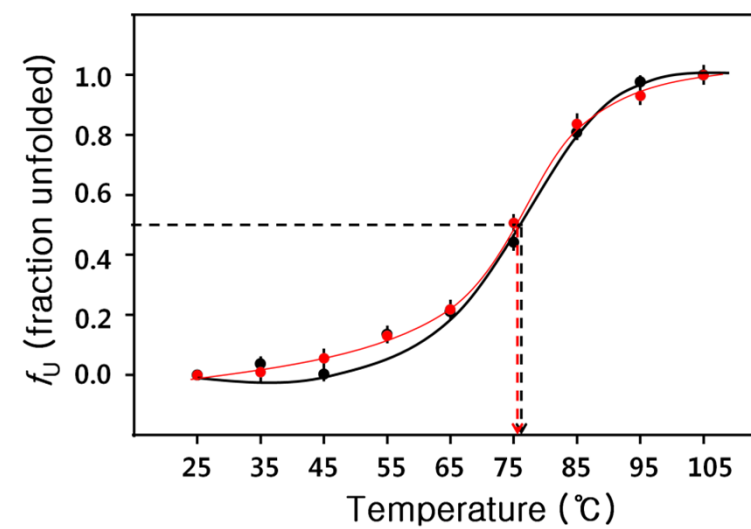

C

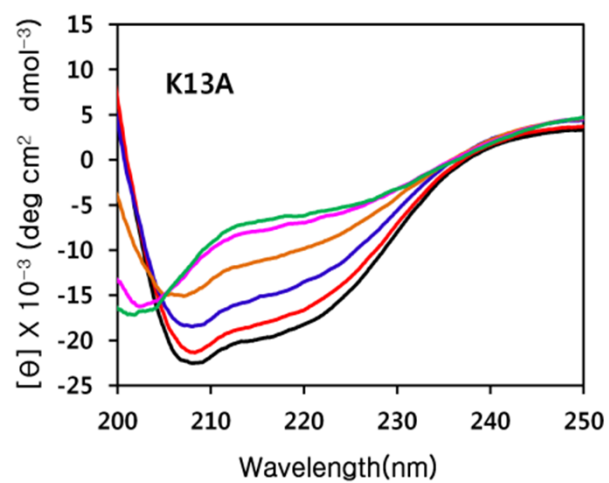

D

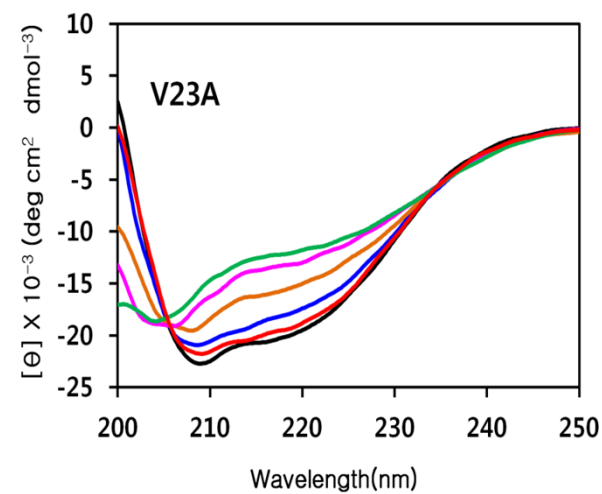

E

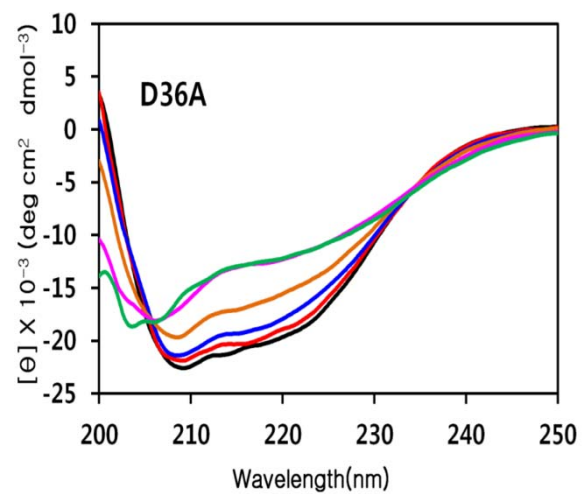

F

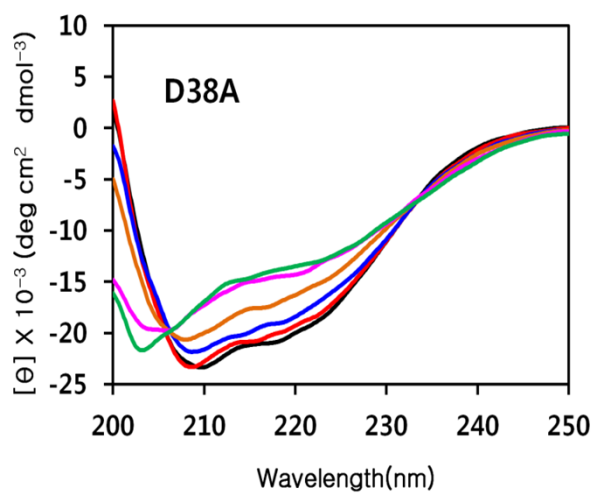

G

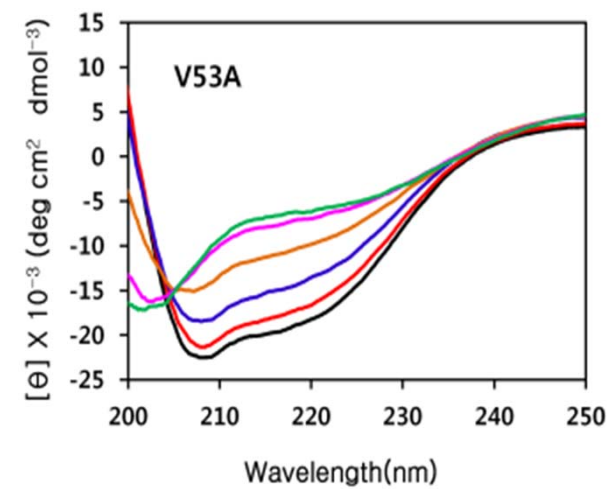

Supplement: S1 File — (A) Far-UV CD spectra of MTH1880 in the presence of Ca2+. Data were acquired at 25°C (black triangle), 45°C (red square), 65°C (blue circle), 75°C (hkaki diamond), 85°C (red triangle), 95°C (green square), and 105°C (red circle). Protein concentration was ~25 μM in a cell of 0.1 mm path length. (B) The fraction of unfolding extracted from far-UV CD spectra at 222 nm with a constant heating rate of 10°C/h as a function of temperature in the presence of Ca2+(red filled circle) and the absence of Ca2+ (black filled circle) are shown by a sigmoidal curve. The transition mid-temperature (Tm) of MTH1880 in the presence of Ca2+ was 75.8 ± 0.5°C. Far-UV CD spectra of MTH1880 mutants in the salt bridge and hydrophobic pocket at temperature values ranging from 25°C to 95°C (C-G). Data were shown at 25°C (black line), 45°C (red line), 65°C (blue line), 75°C (purple line), 85°C (pink line) and 95°C (green line), respectively. (PDF) [file pone.0145853.s001.pdf]
